# The Muslim Headscarf and Face Perception: “They All Look the Same, Don't They?”

**DOI:** 10.1371/journal.pone.0084754

**Published:** 2014-02-10

**Authors:** Umar Toseeb, Eleanor J. Bryant, David R. T. Keeble

**Affiliations:** 1 Bradford School of Optometry & Vision Science, University of Bradford, Bradford, United Kingdom; 2 Division of Psychology, University of Bradford, Bradford, United Kingdom; University of Bologna, Italy

## Abstract

The headscarf conceals hair and other external features of a head (such as the ears). It therefore may have implications for the way in which such faces are perceived. Images of faces with hair (H) or alternatively, covered by a headscarf (HS) were used in three experiments. In Experiment 1 participants saw both H and HS faces in a yes/no recognition task in which the external features either remained the same between learning and test (*Same*) or switched (*Switch*). Performance was similar for H and HS faces in both the *Same* and *Switch* condition, but in the *Switch* condition it dropped substantially compared to the *Same* condition. This implies that the mere presence of the headscarf does not reduce performance, rather, the change between the type of external feature (hair or headscarf) causes the drop in performance. In Experiment 2, which used eye-tracking methodology, it was found that almost all fixations were to internal regions, and that there was no difference in the proportion of fixations to external features between the *Same* and *Switch* conditions, implying that the headscarf influenced processing by virtue of extrafoveal viewing. In Experiment 3, similarity ratings of the internal features of pairs of HS faces were higher than pairs of H faces, confirming that the internal and external features of a face are perceived as a whole rather than as separate components.

## Introduction

It is a cliché of racist discourse that members of some particular ethnic group “all look the same”. It has, however, been shown that persons of one group do tend to find it harder to perform face recognition tasks on stimuli composed of ethnic groups other than their own [1]. This is generally known as the own race bias, and has been investigated in a very large number of studies over the past 45 years or so, employing a profusion of different methodologies including recognition performance and eye tracking (eg [2]).The general finding stands despite the fact that faces of most racial groups are actually of comparable heterogeneity, and this is an effect that seems to be largely independent of the prejudices of the viewer. These findings are of some practical significance, as has been instanced by various cases of miscarriages of justice caused by inter-ethnic misidentification of faces [e.g. 3].

There has been some research looking at the effect of Muslim head/face covering on the recognition of emotions [4], [5], however, what does not seem to have been investigated, is whether wearing the Muslim headscarf (or *hijab*) impoverishes face recognition. There are certainly anecdotal reports of *hijab*-wearing-women being described as more visually homogenous or harder to recognize, but perhaps unsurprisingly people feeling this have rarely expressed themselves in print. In particular, such anecdotes tend to refer to situations in which a woman who has habitually worn the *hijab* unveils and is then frequently not recognized by friends and co-workers, and similarly if a woman starts to wear the *hijab*. This dissimilarity in appearance is illustrated dramatically in a piece of contemporary artwork [6].

As well as being of theoretical interest, such phenomena may have practical implications, as there is some evidence for job discrimination against persons of obviously Muslim appearance [7]. Alleged homogeneity of headscarf-wearing women may also feed into discourses of anti-Muslim prejudice. Since world events such as the attacks of 9/11 and 7/7 there has been a marked rise in anti-Muslim sentiment [8] and Muslim symbols such as the headscarf, beard, minaret, and the Burka are often the target of this.

There are a number of issues to consider when experimentally investigating such effects. It is possible that facial recognition of headscarf-wearing women may be harder for purely perceptual/cognitive reasons. That is, the headscarf removes information about the facial identity (e.g. hair and ears), which may make recognition harder in and of itself. We believe that this is unlikely, as we have shown that recognition for the internal features of faces is as good as for full faces [9]. It may be however, that the presence of the headscarf surrounding a face provides a compelling distraction that reduces performance in at least some participants – such an effect might well be influenced by ethnicity (see Meissner and Brigham [1] for a review of the own race bias) and gender (see Herlitz and Lovén [10] for a review of the own-gender bias). On the other hand, if no effect of the headscarf *per se* is found, it might be that switching the state of hair and headscarf between learning and test (*Switch*) impoverishes performance compared to when the state is consistent (*Same*): we found such an effect when switching between internal features-only faces and full faces [9]. We test these ideas in Experiment 1.

It is possible that any differences in perception between headscarf and full-face stimuli, or between *Same* and *Switch* conditions are due to relatively low-level eye movement strategies. In order to investigate this, in Experiment 2 we measured the proportion of fixations to external and internal features for both hair and headscarf stimuli, in both *Same* and *Switch* conditions. If similar results are found for headscarf stimuli as were found for internal-only stimuli, it would be tempting to conclude that the only role of the headscarf in face perception is to remove external features. However, it is quite conceivable that headscarf-wearing faces actually appear more similar, without affecting recognition performance (it has been shown, for example, that the presence of a *hijab* modulates the perceived attractiveness of female faces [11]), so we investigated this in a rating experiment (Experiment 3).

## Experiment 1

As it is thought that switching the external features between the learning and test stage might affect the holistic representation of faces [9], in the following section, different phenomena which investigate this holistic basis for face processing are briefly mentioned. Yin [12] investigated the Face Inversion Effect which showed that upright faces are recognised better than inverted faces. Many researchers [12]–[17] have concluded that upright faces are processed holistically, whereas inverted faces are processed in more of a featural manner. Further evidence for the holistic nature of face processing comes from the composite face effect (when the top half of a very familiar face is combined with the bottom half of an unfamiliar face it is perceived as a new face) [18], [19] and the whole-part superiority effect (parts of the face are better recognised in the context of the whole rather than in isolation) [20]. However, in some of these studies, hair was removed.

Ellis et al. [21] conducted experiments in which a yes/no recognition paradigm was employed. During the learning stage participants viewed a series of faces that were presented with hair. Then at test, the participants were divided into three groups and asked to decide which faces they had previously seen (faces at test were either with internal and external features, only internal features, or only external features). These researchers found that performance was best when participants viewed the whole face at test compared to the other two conditions. There was no difference in the recognition of unfamiliar faces in the group which viewed only internal features when compared to the group which viewed only external features. Ellis et al. [21] concluded that internal and external features play an equal role in the processing of unfamiliar faces. Wright and Sladden [22] also investigated the role of hair in face recognition using a yes/no recognition task. Half of the faces were learnt with hair and the other half were learnt without hair. Then in the test stage, all the faces were presented with hair. These researchers found that performance was higher when hair was present at learning compared with when it was not. Both these studies [21], [22] took this as evidence for the importance of hair in face recognition. However, we conducted a similar experiment [9] in which participants learnt faces with and without hair and were tested on faces for which the external features were congruent or incongruent to those at learning. We found that there was no difference in the recognition of faces with and without hair when the external features remained the same between learning and test, however, when the external features were switched, there was a drop in performance. We concluded that there is sufficient information in the internal features of a face for optimal recognition in a yes/no recognition task and that the importance of hair varies with the demands of the task. The aim of the first experiment here was to investigate if the same effect would be found using headscarf stimuli or whether the headscarf would act as a distracter or otherwise reduce performance. Research that investigates the Muslim headscarf and its role in face perception is limited to a small number of studies. Megreya and Bindemann [23],[24] investigated the Muslim headscarf using a face matching task. They found that Egyptian participants were able to match unfamiliar faces better from internal compared to external features, however, British participants were able to match faces better with external features than internal ones. These researchers attributed this “internal feature advantage” amongst Egyptian participants to perceptual expertise as most women in Egypt wear a headscarf. The findings of Kret and de Gelder [4] and Fischer et al. [5] that the presence of the headscarf or the niqab can affect the perception of emotion makes it plausible that there could be a similar effect on recognition performance. Furthermore, the mass of evidence concerning the own race bias makes it plausible that there might be different perceptual effects of Muslim dress on different groups. Additionally, given that an own gender bias has been reported in previous research, (see Herlitz and Lovén [10] for a review), gender will be controlled for in Experiment 1.

### Material and Methods

#### Ethics

All of the experiments that are reported in this paper have been approved by The Biomedical, Natural and Physical Sciences, University of Bradford, Research Ethics Panel. All participants provided written informed consent.

#### Participants

A total of 84 participants took part in Experiment 1 (36 males & 48 females) with a mean age of 22.52 years (SD = 5.70). Participants were 18 South Asian males (mean age = 20.11 years, SD = 1.88), 18 White males (mean age = 23.61 years, SD = 4.15), 18 White females (mean age = 27.83 years, SD = 9.19), and 30 South Asian females (mean age = 20.13 years, SD = 1.88).

#### Stimuli

**Figure 1:**
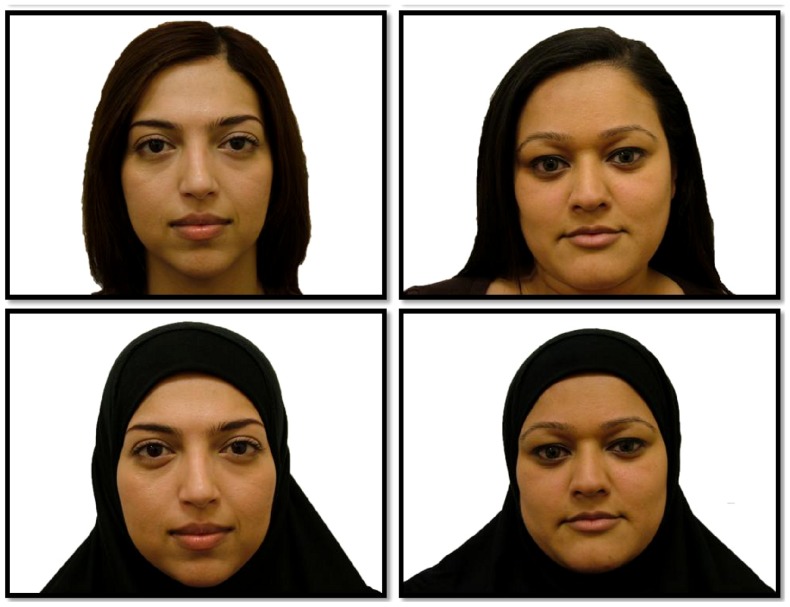
Examples of faces either with hair or with headscarf.

All the stimuli used in our experiment were images of South Asian Females (for examples, see Figure 1). Our research was part of a wider project looking at the effect of the Muslim headscarf on face recognition. For this reason, the stimuli used in our experiment were only South Asian Females. A total of 24 South Asian females between the age of 18 and 30 years were photographed twice. The first photograph was taken with the participant's hair showing (H) and the second wearing a Muslim headscarf (HS). The colour photographs were 1280 pixels×960 pixels with a 32-bit depth. All photographs were then programmed into the E-prime software [25],which was used to run the experiment. Participants gave written informed consent, as outlined in the PLOS consent form, to publication of their photograph.

#### Design

A mixed-subjects design was employed in which the between-subjects variables were Gender (Male or Female), Race (South Asian or White), and Condition (Same* or Switch**). The within-subjects variable was State of Stimuli at Test (Hair and Headscarf). Participants only took part in **one of the two** conditions but saw both types of stimuli within each condition (Hair and Headscarf)

*“*Same*” refers to the condition in which the stimuli remained the same between the learning and test stage. In this condition participants viewed H and HS faces intermixed in the learning stage. Later in the test stage they were presented with the same stimuli plus distracter faces which had not previously been viewed. The distracter faces were both HS and H. The participants in this condition took part in H→H and HS→HS trials. In general, we use the nomenclature “X→Y” to indicate that the stimulus was in state X at learning, and state Y at test (because in the subsequent condition the stimuli change between the learning and test stage).

**“*Switch*” refers to the experimental condition in which the stimuli were switched from the learning to the test stage. In this condition participants viewed both H and HS faces intermixed in the learning stage. At test, the external features of previously seen faces were switched. That is, faces that were viewed with hair in the learning stage were now presented with a headscarf and *vice versa*. The participants in this condition took part in H→HS and HS→H trials.

In order to prevent coincidental differences in recognisability of the faces producing a spurious difference in performance between, say, the H→H and HS→HS trials, a form of counterbalancing was employed. For this condition half of the participants would see half of the faces in the H form, with the other half being seen in the HS form. The other half of the participants would see the faces in their complementary forms. In this way each stimulus actor would be seen an equal number of times in each state.

#### Procedure

All participants were given 8 practice trials followed by the main experiment in which participants were presented with 12 pictures in the learning stage (hair and headscarf intermixed); each for 6000 ms with an inter-stimulus interval of 1000 ms which was followed by a distracter task (word search). At test, participants were presented with 24 pictures (12 previously seen faces and 12 distracter faces) and were required to decide which ones they had previously seen. Each face was presented for 5000 ms after which a blank screen appeared until the participant responded.

### Results

**Figure 2:**
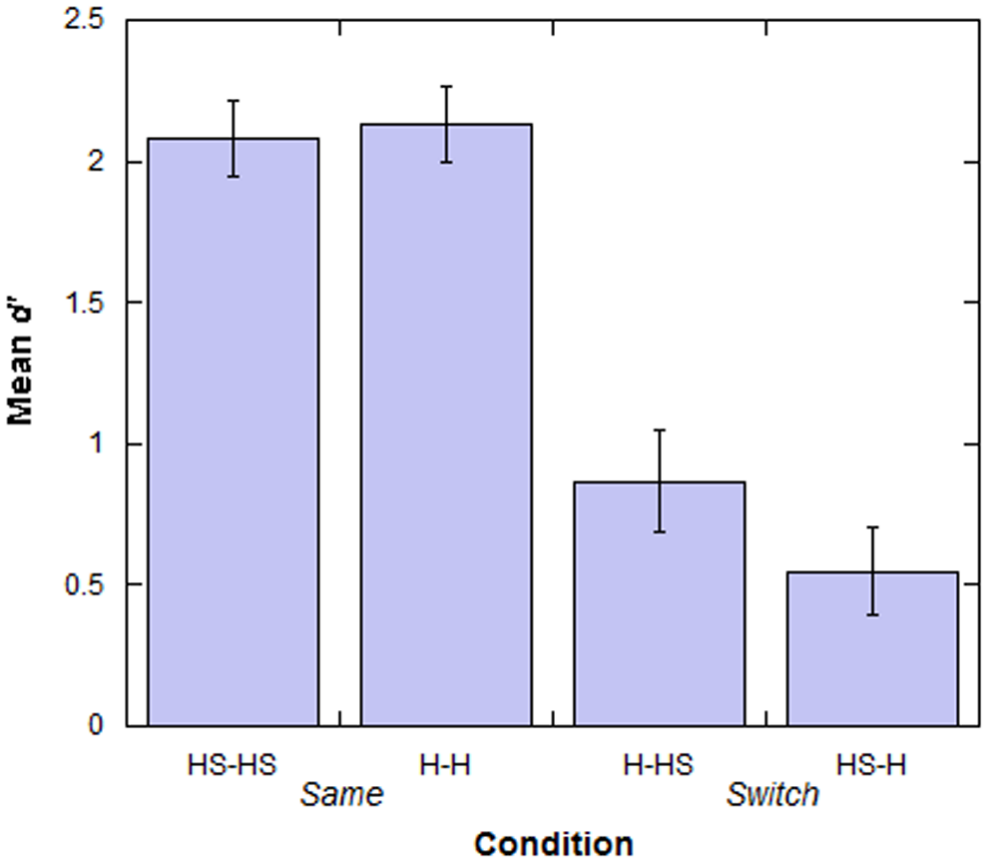
Performance levels in the *Same* and *Switch* conditions for Experiment 1 split by the type of trial in each condition. Error bars represent Standard Error. HS-H, (for example), refers to those trials in which the faces that were presented as HS faces in the learning stage were switched and presented with H in the test stage.

Participants' sensitivity scores, *d′* [26], were put into a four-way mixed ANOVA (Gender×Race×State of Stimuli at Test×Condition). The analysis revealed a significant three-way interaction between State of Stimuli at Test×Gender×Race (F (1, 76) = 4.07, p = 0.047, partial η^2^ = 0.051), as shown in Table 1. This interaction was investigated further with a series of 2×2 ANOVAs. First the data was split by Gender. State of Stimuli at Test was entered as a within subjects variable and Race was entered as the between subjects variable. There were no significant main effects or interactions for either Males (p>0.05) or Females (p>0.05). Next the data was split by Race. State of Stimuli at Test was entered as the within subjects variable and Gender was entered as the between subjects variable. There were no significant main effects or interactions for either South Asian (p>0.05) or White participants (p>0.05). Finally, two between subjects ANOVAs were conducted: one for Headscarf at Test and one for Hair at Test. For both of these ANOVAs, Gender and Race were entered as the between subjects variables. No main effects or interactions were observed for either the Headscarf at Test faces (p>0.05) or the Hair at Test faces (p>0.05). A main effect of Condition was also observed (F (1, 76) = 74.086, p<0.001). This showed that participants in the *Same* condition performed significantly better compared to those in the *Switch* condition. There were no main effects of Gender, Race, or, crucially, State of Stimuli at Test. These results are shown in Figure 2.

**Table 1:** Mean *d′* and Standard Deviation divided by Gender, Race, and State of Stimuli at Test.

**Gender**	**Male**				**Female**			
**Race**	**South Asian**		**White**		**South Asian**		**White**	
**Stimulus at Test**	**HS**	**H**	**HS**	**H**	**HS**	**H**	**HS**	**H**
Mean *d′*	1.51	1.53	1.53	1.01	1.88	1.61	1.12	1.54
Standard Deviation	1.04	1.48	0.81	1.12	1.28	1.05	1.24	1.26

HS represents headscarf and H represents hair. The data is collapsed across the two conditions.

### Discussion

Experiment 1 yielded two key findings: firstly, when the state of the stimuli remained the same between the learning and the test stage (*Same*), there was no difference in the recognition of the stimuli presented with hair and with headscarf. This is revealed by the lack of interaction between Condition and State of Stimuli at Test. This supports the notion that in these experimental conditions there is sufficient information in the internal features of a face for optimal processing to occur and that the headscarf did not act as a distracter. Secondly, however, when the stimuli were switched from learning to test (*Switch*), performance was significantly worse. We suggest that in the *Switch* condition, holistic processing mechanisms were disrupted, and thus these findings provide further evidence for the holistic nature of face processing. These results are in accord with our previous findings [9], which were obtained using hair and cropped stimuli.

Although a statistically significant three-way interaction was observed between State of Stimuli at Test, Gender, and Race, this was not elucidated by posthoc analyses. Additionally, there appeared to be a weak differential trend for Males and Females in their performance but, again, there was no statistically significant difference between the Genders. These unstable effects could be considered and investigated in future work.

These results are supportive of previous work [21], [22] in which it was found that participants were worse at face recognition when the state of the stimuli was switched between learning and test. However, these researchers attributed the difference to the importance of hair. Instead, as in our previous work [9], we maintain that the difference is due to the incongruence of the external features between the learning and test stage.

To investigate the role of the Muslim headscarf further, Experiment 2 used eye-tracking methods to measure eye-movements during a yes/no recognition task in which both *Same* and *Switch* conditions were employed. The aims of Experiment 2 were to investigate a possible cause for the drop in performance between the *Same* and *Switch* conditions and between H and HS and also how eye-movements vary by task demand. That is, as the findings from Experiment 1 showed that the importance of hair in face recognition varied depending on the task at hand (*Same* or *Switch*), it may be that eye-movements change according to the circumstances. For example, learning faces may tap into different perceptual mechanisms compared to the recognition of faces, therefore, a difference in the eye-movements between the two stages may be expected.

## Experiment 2

Eye movements are clearly an important aspect of visual perception, and the patterns of fixations and saccades in response to face stimuli have been extensively studied for many years [e.g. 27]. Specifically, when investigating the role of eye-movements during a yes/no recognition task, it has been found that in the trials in which participants' eye movements were restricted during the learning stage, they performed significantly more poorly than if they were able to freely learn the faces [28]. More generally, the eye movements exhibited by a person viewing a face may reveal aspects of the underlying processing. One fairly general finding of relevance to our study is that in most cases a very high proportion of fixations are to internal facial features [29]–[33]. In the *Switch* condition in Experiment 1, the external features were affecting performance, so one possibility is that in this experiment there were actually many fixations to the hair or headscarf. This could be during both learning and test, or it could be that the change in external features triggers a change in patterns of eye movements – possibly extensive scanning of the changed external features. A further possibility is that eye movements are actually very similar for *Same* and *Switch* conditions.

We believe that the drop in performance when the stimuli were switched to/from headscarf compared to when they remained the same was due to the disruption of holistic processing mechanisms at some level. There is evidence that eye movements can alter for different face processing strategies, such as featural and holistic processing [34]. Furthermore, it has been found that the decrease in recognition performance for inverted faces, which is thought to be the signature of disrupted holistic processing, is accompanied by changes in fixation patterns [35], although other work did not discover any such changes in a similar experiment [36]. Chan and Ryan [37] found that altering the length and style of hair on computer-generated faces did affect eye movements in that previously unseen faces had similar eye movement patterns to manipulated faces (the equivalent of switched faces in our experiments). Hence, it is eminently possible that eye movements may be altered in our *Switch* condition, although the mixed evidence precludes a definite prediction of this.

There is also the possibility that Hair and Headscarf stimuli are themselves associated with different eye movements, despite producing the same behavioural response, so we compared them. In short, we investigated eye movement patterns for subjects performing the same task as in Experiment 1 in order to see if our results could be explained by this aspect of visual processing. As we are investigating the role of external features in face recognition, the primary measure studied was the proportion of fixations to external features.

### Materials and Methods

#### Participants

A total of 41 participants took part in this experiment (mean age = 22.90 years, SD = 3.93). There were 10 South Asian males (mean age = 22.40 years, SD = 7.09), 11 South Asian females (mean age = 20.73, SD = 1.27), 10 White males (mean age = 24.90, SD = 5.45), and 10 White females (mean age = 23.80, SD = 4.24). All participants had normal or corrected-to-normal vision.

#### Stimuli

**Figure 3:**
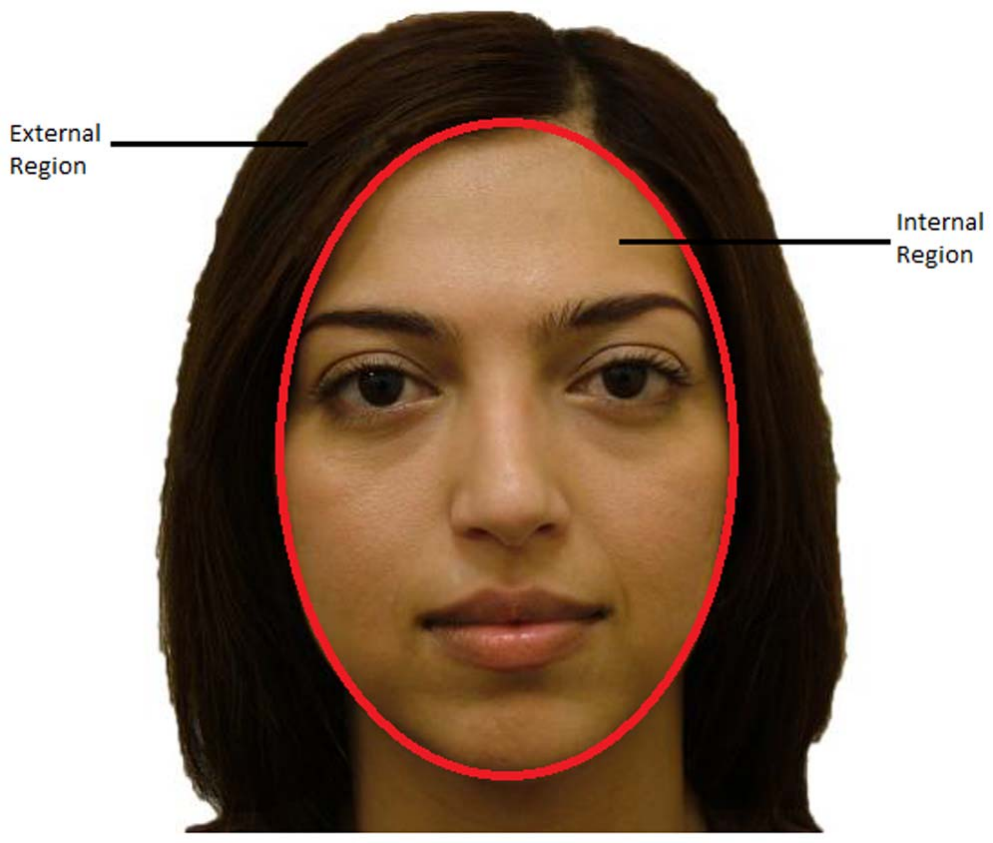
Illustrating the internal and external regions.

The stimuli used in this experiment were the same as the previous experiment. The viewing distance was kept constant at 60 cm and the mean visual angles of the faces were 10.94° horizontal and 14.72° vertical. The stimuli were displayed using MATLAB Version 7.6.0. The regions classified as internal and external are shown in Figure 3.

#### Apparatus

A Cambridge Research Systems (CRS) eye tracker was used (sampling frequency = 50 Hz, resolution = 0.1° and, accuracy <0.5°). The CRS eye tracker was controlled by the Video Eye Tracker MATLAB Toolbox Version 1.26 which was integrated with CRS ViSaGe.

#### Procedure

Participants followed a similar procedure to the one reported in the previous experiment but, with some minor differences. Viewing was binocular, however, only one eye was tracked (as in [32]). Participants performed a 25 point calibration prior to commencing the practice trials, the learning stage, and the test stage.

### Results

#### Sensitivity *(d′)*

**Figure 4:**
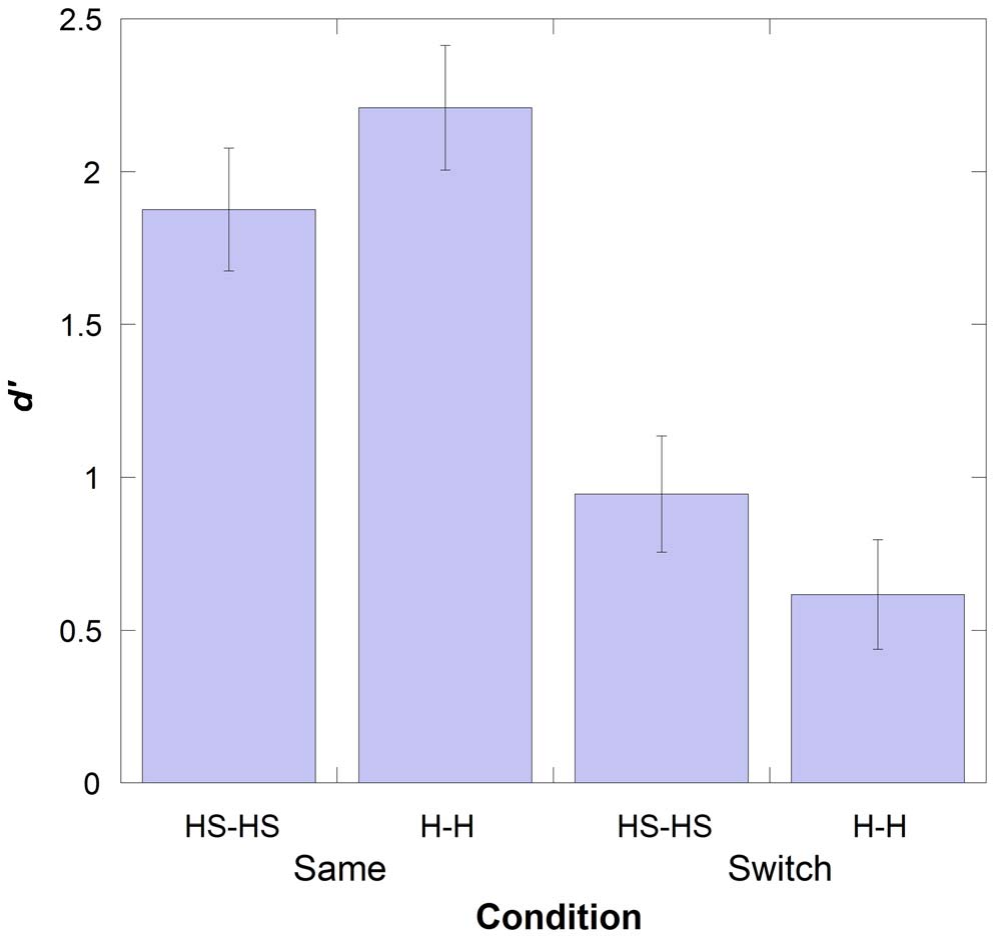
Performance levels in the *Same* and *Switch* conditions for Experiment 2 split by the type of trial in each condition. Error bars represent Standard Error. HS-H, (for example), refers to those trials in which the faces that were presented as HS faces in the learning stage were switched and presented with H in the test stage.

Although Gender and Race were controlled for when recruiting participants, they were not included in the analysis because Experiment 1 did not find an effect of these variables. Thus, a two-way mixed ANOVA was conducted in which Condition (*Same* or *Switch*) was entered as the between-subjects variable and Stimuli at Test (Hair and Headscarf) was entered as the within-subjects-variable. A main effect of Condition was observed, F (1, 39) = 34.74, p<0.001, partial η^2^ = 0.471. This showed that participants in the *Same* condition performed significantly better than the participants in the *Switch* condition (difference in *d′* = 1.26), as in Experiment 1. This is shown in Figure 4.

#### Percentage Fixations on External Features

Next, the percentage of fixations on external features was investigated. The internal and external regions are shown in Figure 3. This was calculated relative to the total number of fixations so that, percentage of fixations on internal features+percentage of fixations on external features + percentage of fixations outside defined internal or external regions = total number of fixations. In fact, the proportion not on either external or internal regions were extremely small, (less than 1.5% in the learning stage and less than 0.5% in the test stage) and we do not believe that their inclusion or exclusion would affect the analysis. In the test stage, only data from faces that were shown in the learning stage was used (previously seen faces) which was consistent with the analysis in previous work [29].

Firstly we investigated whether the drop in performance between the *Same* and *Switch* conditions was due to the difference in the fixations on the external features during the test stage. To do this, participants were divided into two groups, those that fixated on external features during the test stage and those that did not. We then conducted a Fischer's exact test. The independent variable was entered as Condition (*Same* or *Switch*) and the dependent variable was entered as Fixations on External Features (Yes or No). There was no significant relationship between Condition and whether participants fixated on the external features during the test stage (p = .085). Thus, whether participants fixated on the external features did not differ between the *Same* and *Switch* condition.

Next, a Wilcoxon sign rank sum test was used to investigate whether participants fixated more on the external features when presented with hair stimuli compared to the headscarf stimuli, for both the learning and the test stage. It was found that, in the learning stage, participants fixated more on external features in the hair stimuli compared to the headscarf stimuli (z = −4.547, p<.001), however, no such difference was found in the test stage (z = 1.552, p = .121). Thus, regardless of Condition (*Same* or *Switch*), participants fixated more on external features in the learning stage, when hair was available, than when a headscarf was available.

### Discussion

The most salient feature of our results is that there were relatively few fixations to external regions in all conditions. This is in line with the bulk of earlier studies [29]–[33] on the subject. However, in this case it is a finding that has added interest, as the external features are clearly being processed sufficiently to impair performance in the *Switch* condition. Given the rather large drop in performance shown in Experiment 1 (approx 20%) and the very small proportion of external fixations at test (∼1%), it suggests that foveal processing of external features is not a requirement for them to impact on face recognition mechanisms – a finding which does not seem to have been reported before.

When hair was visible during the learning stage, participants inspected it more than they did the headscarf, although still at only 8% of fixations. However, no such difference was found at recognition. Past work has found that as image resolution of famous faces decreased, the importance of external features increased [38]. Similarly for our stimuli (which are unfamiliar), it may be that hair is processed and stored as a reserve for instances when the task is more difficult. Additionally, it may be that the presence of a headscarf shifts the attention towards the internal features which causes the participants to encode them more efficiently, resulting in the lack of difference in sensitivity for headscarf and hair stimuli.

The most surprising finding was that whether a participant fixated on the external features was not predicted by Condition (*Same* or *Switch*). This is despite the fact that performance was worse in the *Switch* condition, which must in some sense be due to the external features. Presumably the disruption to performance occurs at some level distinct from that which generates eye movements. This is a similar result to that of Williams and Henderson [36] who found no change to eye movements when holistic processing was disrupted by inversion, but is rather different to a number of other studies [34], [35], [37] in which it was found that various aspects of eye movements were affected by changes to face processing mechanisms. Evidently the relationship between eye movements and holistic processing is a complex one. The key point is that in this particular task eye movements (or specifically proportion of external fixations) do not appear to be involved in the drop in performance for *Switch* stimuli.

So if eye movements are not affected, and the external features are only rarely fixated, how then do the hair and headscarf exert their influence on face perception? The processing of faces may be thought of as an integrative process in which the internal features and external features are processed together as a whole rather than separately. For this reason, participants may find it difficult to completely ignore the external features. Therefore, we wished to explore the effect of explicitly instructing participants to ignore the external features yet still making them visible, in order to see whether the visual system is still affected by their presence. The next experiment investigates this concept in a task in which participants were asked to rate the similarity of pairs of faces.

## Experiment 3

Some people think that headscarf-wearing females are more visually homogenous than non-headscarf wearing females. Goldstein and Chance [39] investigated a somewhat similar issue, but found that there was no difference in the number of instances of when pairs of Japanese faces were rated as more similar compared to when White American pairs were perceived as being more similar, when being viewed by participants of either race. This implies that Japanese faces are actually equally as perceptually homogenous as White American faces. This study shows that the attribution of homogeneity to another race is not apparent at the level of visual perception. This was despite the fact that according to anecdotal evidence and verbal reports from participants in this study, when commenting on why they thought recognition of the other race was not as good as their own, participants often stated “they all look alike to me”.

To understand the nature of holistic processing in such a task, Popivanov and Mateeff [40] conducted an experiment in which they presented participants with pairs of faces which were either inverted or upright. Participants rated inverted faces as more similar to each other compared to upright faces. This demonstrates that as the task becomes more difficult or as the holistic processing of faces is disrupted, they tend to look more alike.

Participants in Experiment 3 were presented with pairs of faces which either: both had hair, both had a headscarf, or one had hair and the other had a headscarf, and they were asked to rate how similar they thought the **internal features** of the two faces were. Based on the evidence from the previous experiments, it was predicted that, although participants will not look directly at the external features (and try to ignore them), they will play some role in the similarity rating of the internal features.

### Materials and Methods

#### Participants

A total of 32 participants (mean age = 22.25 years, SD = 3.36) took part in this experiment. They were eight South Asian males (mean age = 20.88, SD = 1.55), eight South Asian females (mean age = 21.88, SD = 3.52), eight White males (mean age = 23.25, SD = 4.30), and eight White females (mean age = 23.13, SD = 3.40).

#### Stimuli

The stimuli that were used in this experiment, both headscarf and hair, were the same as in the previous experiments.

#### Design

A mixed-subjects design was used in which participants were divided into three groups. Participants in each group viewed eight faces which were presented both with hair and with a headscarf, therefore resulting in a total of 16 images. These 16 face images were compared to each other resulting in a total of 136 pairs, which were presented side by side, and each viewed once by the participant. The difference between the three groups was the particular sets of face stimuli that were used. The within-subjects variable was the type of pair (Headscarf*, Hair**, & Mix**). In the Mix trials the headscarf face always appeared on the right hand side of the pair.

* Headscarf refers to the pairs of faces in which both of the stimuli were displayed with a headscarf.

** Hair refers to the pairs of faces in which both of the stimuli were displayed with hair.

*** Mix refers to the pairs of faces in which one of the stimulus faces was displayed with a headscarf and the other was displayed with hair.

#### Procedure

Participants were presented with pairs of faces which they were required to rate on a scale of 1 to 7, based on how similar they perceived the **internal features** of the two faces to be, 7 being ‘very similar’ and 1 being ‘not similar at all’. Participants were advised by on-screen and verbal instructions to make their judgements based solely on the internal features. They were then shown a picture of a sample face with the external features cropped out to make sure that they understood what is meant by “only internal features”. Participants each viewed 136 pairs of faces and were advised to use a variety of keys between 1 and 7.

### Results

Participants' data was divided into three types: mean similarity rating for Hair pairs, mean similarity rating for Headscarf pairs, and mean similarity rating for Mix pairs. The data from the three groups of participants was collapsed to form one dataset.

#### Similarity Ratings

**Figure 5:**
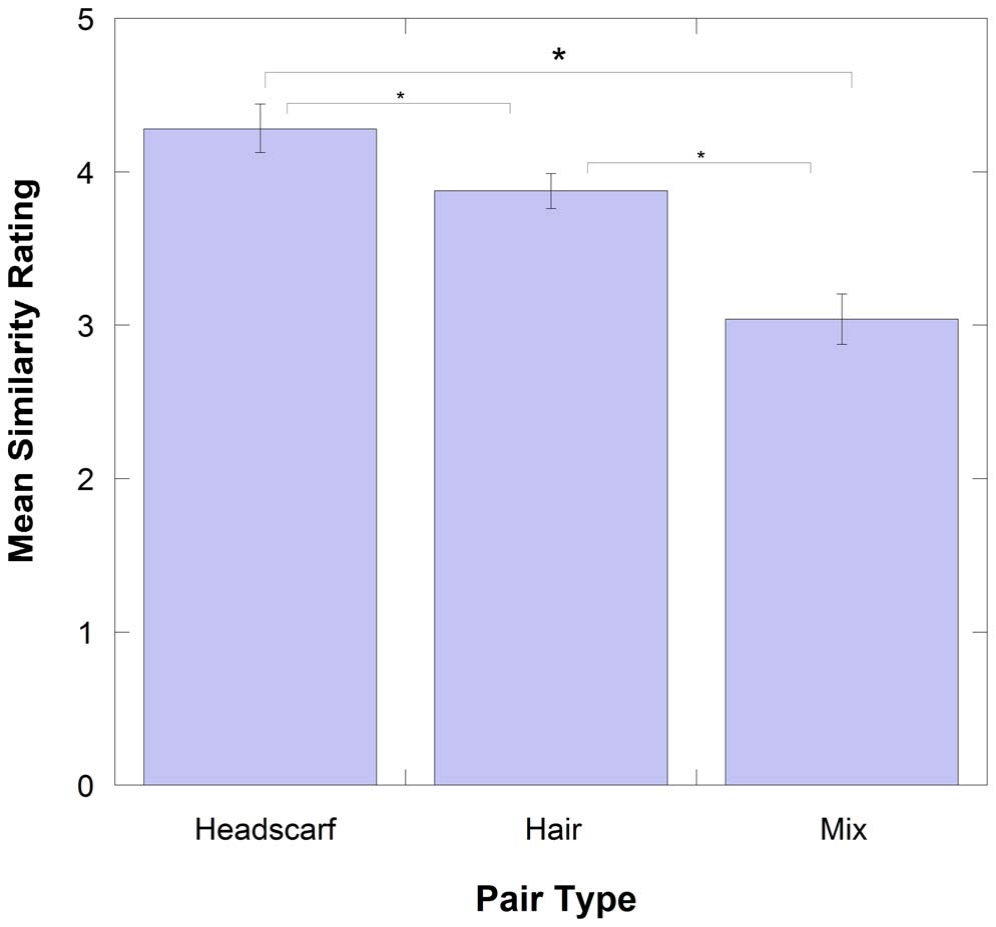
Mean similarity ratings for the different types of pairs. Error bars represent Standard Error. * is significance at p<0.001.

A repeated measures ANOVA yielded a main effect of Type of Pair (F (1.28, 39.65) = 61.554, p<0.001, partial η^2^ = 0.665). Bonferonni pairwise comparisons showed that each of the pair types were significantly different to the others (Hair-Headscarf (p = 0.001), Hair-Mix (p<0.001), and Headscarf-Mix (p<0.001)). These differences are demonstrated in Figure 5.

#### Similarity Ratings for Same Pairs

Next, the analysis was conducted on those particular trials in which both of the images in the pair were of the same person. These trials conducted were of three types: **same person** with hair in both images (Both Hair), **same person** with headscarf in both images (Both Headscarf), and **same person** with hair in one image and headscarf in the other image (Headscarf & Hair). The mean rating given to the ‘Both Hair’ images was 6.87 (SD = 0.55) and mean rating given to the ‘Both Headscarf’ images was 6.85 (SD = 0.54). A One-Sample t-test was conducted for both these comparisons to confirm that they **were not** significantly different from 7, which was the maximum possible similarity rating (Both Hair (t (31) = 1.37, p>0.05) & Both Headscarf (t (31) = 1.55, p>0.05)). However, the mean rating given to the ‘Headscarf & Hair’ images was much lower at 5.64 (SD = 1.28). Again, a One-Sample t-test was conducted which showed that this score **was** significantly different to 7 (t (31) = 6.04, p<0.001).

Paired sample t-tests were conducted on these figures and it was found that ‘Headscarf & Hair’ images were rated as significantly less similar that ‘Both Hair’ (t (31) = 5.304, p<0.001) and ‘Both Headscarf’ images (t (31) = 5.425, p<0.001).

### Discussion

Evidently, the internal features of headscarf-wearing faces are perceived to be more homogenous compared to faces with hair. One of the reasons for this may be that, although participants were instructed not to look at the external features they found them difficult to ignore when comparing the internal features (or at the very least they processed them at some level).

Participants were advised both in the written and verbal instructions to make their judgements based on only the internal features. If it was possible to selectively process only the internal features of a face, then there would be no difference between the three different types of pair. However, it was clearly not possible to separate the internal from the external features, and an explanation for this is that in general during the perception of faces, humans are not able to separate the different parts of the face. Instead, a holistic representation of the faces is created, which is then used to match with the corresponding face. This is quantified by the finding that, when a pair consists of two faces with the same internal and external features (both faces with hair or both faces with a headscarf), the similarity judgements were not significantly different to 7 (very similar) for headscarf and hair pairs. However, when the same internal features were presented with different external features (one hair and one headscarf) then similarity ratings differed compared to when the internal and external features were the same in both faces of the pair. This demonstrates that participants actually use external features (to some extent) to determine the similarity between two faces. Together with the previous findings (Experiments 1 & 2), these results show that holistic processing is involved with not only memory for human faces, but also the perception of faces. That is, holistic processing occurs at the level of perceiving the stimuli and is not just a product of memory representations. Furthermore, these findings show that the different regions of a face are processed interactively and cannot simply be parsed into internal and external features. These findings may aid in the understanding of why a drop in performance was observed in the yes/no recognition task between the *Same* and *Switch* condition. It may be that, because the face is **perceived** as whole, it is represented and stored in memory as a whole (independent of the internal/external feature distinction). Therefore, in the *Switch* condition, when the stimulus presented at test did not match the mental representation, the participants were unable to establish that only the external features had been amended, rather, it was perceived as a previously unseen face which led to a lower sensitivity score.

A limitation of the design of Experiment 3 was that, in the Mix trials, the headscarf face always appeared on the right hand side of the pair. It would be interesting for future studies on this topic to replicate this study whilst counterbalancing the location of the headscarf face (left & right), thereby obviating the possibility of the right hemisphere advantage. We feel that such an effect is highly unlikely to have affected our main conclusions for two reasons; firstly, the effect that we have observed is large and therefore, any hemispheric advantage would have to be very strong to nullify this effect. Secondly, to our knowledge, the literature around hemispheric advantages with regards to faces focuses on their recognition. As the task in Experiment 3 was to compare faces, we believe that it is unlikely that a hemispheric advantage would exist in such a task.

## General Discussion

We have shown that the mere presence of a headscarf, as opposed to hair, does not impoverish face recognition, thereby confirming and extending our previous findings [9] that *removing* the hair does not decrease performance. When the state of the external features changed between learning and test faces however, performance did get much worse, as we previously found when switching between hair and cropped stimuli [9]. Thus, the effect of the headscarf on face recognition seems to be assimilable to a view of the role of external features as only affecting recognition *performance* if there is a change to them. There is a range of experimental evidence which is also consistent with this view. The results of two previously discussed studies [21], [22] can be interpreted in this light, although the authors did not do so. The effect of changing hair styles appears to be similar [37], [41], although others also found an effect on eye movement patterns [37], which we did not. There is also some evidence that changing tattoo patterns [42] or makeup [43] can affect recognition performance, although these are not external features. Our findings are compatible with the concept that a large part of face perception consists of holistic face processing – different face parts being perceptually melded to form a unified percept. There is a body of fMRI imaging work, mostly concerned with face adaptation, that is consistent with this idea [44]–[46].

Future work could investigate the same effect but use White or Black females as stimulus faces as well as the South Asian ones. It may be that we have grown accustomed to seeing South Asian faces with a headscarf which is why, in general, the headscarf does not affect recognition. Additionally, future work could adopt a modified yes/no paradigm where the learning and test images are slightly different, thereby obviating the possibility of image matching strategies being used in both Experiment 1 and 2, although as Sporer [47] points out, this latter methodology is only rarely employed. Moreover, even though in the experiments reported here hair and headscarf styles were tightly controlled, future work could use stimuli with a wider variety of hair and headscarf styles and colours. Again, we believe that this would most likely not affect our main conclusions because the stimuli type was tightly controlled and the faces were counterbalanced.

Although the headscarf does not in general affect recognition, we have shown in Experiment 3 that it *does* affect appearance, in the sense that women wearing it are deemed to look more similar than women with hair, who in turn appear more similar than a heterogeneous pair of women. It is important to note that this effect does not depend on race or gender, or, as far as we can tell, on the headscarf-wearing status of the viewer. So we have not found an analogue of the Own Race Bias, but rather a cognitive or perceptual effect on processing more similar to masking effects than anything else. In a sense then, although one cannot say “they all look the same”, one could say that “many of them look quite similar – and to people of varying backgrounds”. In contrast, Megreya and Bindemann [23] did find that the nationality of their participants affected the extent to which internal and external features were used in a face matching task, implying that they may have been tapping a different mechanism.

Conversely to the increased similarity ratings of women wearing headscarves, the same woman can appear very different when wearing a headscarf or with hair, as evidenced by the failure of the subjects in Experiment 3 to rate the internal features of the same woman as having a similarity at the highest level when the faces had different external features. These effects, combined with the *Switch* condition in Experiment 1 would seem to explain the anecdotal reports of apparent similarity of headscarf-wearers and non-recognition of women who change from hair to headscarf or vice-versa with which we began this paper, but would not explain failures to recognize headscarf wearers.
